# Factors influencing the behavioral intention to use contactless financial services in the banking industry: An application and extension of UTAUT model

**DOI:** 10.3389/fpsyg.2023.1096709

**Published:** 2023-03-10

**Authors:** Lu Chen, Jing Jia, Chengzhen Wu

**Affiliations:** ^1^School of Business, Changzhou University, Changzhou, China; ^2^School of Business, Hanyang University, Seoul, Republic of Korea

**Keywords:** contactless financial services, banking industry, unified theory of acceptance and use of technology model, trust, perceived risk, perceived advantage

## Abstract

**Introduction:**

Contactless financial services are an innovative exploration of the banking industry to integrate digital technology. This study further modified the UTAUT model based on the theories of trust, perceived risk, and perceived advantage and constructed a conceptual model to examine the influencing factors of the behavior of using contactless financial services. The purpose of this paper is to figure out factors influencing users’ behavior intention of contactless financial services, in order to promote use behavior, and boost the further development of contactless financial services.

**Methods:**

The model was validated using the data collected through questionnaires. The structural equation modelling (SEM) method was used to validate the research model. We analyzed the generated hypotheses by using AMOS version 23.0. In this study first analyzed the measurement model to test the reliability and validity of the instrument, and then analyzed the structural model to test our research hypotheses.

**Results:**

The results reveal that trust and perceived risk are important factors that affect the behavioral intention of contactless financial services; users perceive that contactless financial services have advantages over traditional offline channel, their intention to use contactless financial services will be increased; social influence also has a positive impact on behavioral intention.

**Discussion:**

This paper not only provides a theoretical understanding of contactless financial services use behavior but also offers practical insights to government legislative branches and app developers. By providing personalized services and refining the policies and regulations in the digital environment to promote the development of contactless financial services.

## 1. Introduction

The COVID-19 pandemic, as a public health emergency, occurred at a turning point of the great changes in globalization and has had a profound impact on the global economy and society. Under the influence of the pandemic, labor-intensive, contact industries have encountered serious operational crises. New forms of the contactless economy (e.g., intelligent logistics, online education, e-government, and telecommuting) supported by digital technologies have emerged during the fight against the pandemic (Ping, 2020). In the coming period, the new business model of contactless services will replace the traditional business model more rapidly and play a role in the transformation and upgrading of industrial and economic structures. In such a context, the banking sector, as a provider of financial services, needs to be further integrated with digital information technology to accelerate the digital transformation of the industry and improve the ability to provide contactless financial services (Ba and Liu, 2020).

Contactless finance is not an unfamiliar concept. American scholars introduced the concept of service encounter at the end of the 20th century and considered it the interaction process between customers and employees. With the development of technology, new services such as self-service devices and Internet services have gradually emerged, drawing attention and being advocated. Thus, intangible encounters have become an integral part of service encounters. In this study, contactless finance refers to the contactless operation of financial institutions in providing users with financial services such as payment and settlement, wealth management, and asset financing through telecommuting and online operations.

Contactless financial services are a part of the digital financial innovation in China’s banking industry due to the changing business environment. At a certain stage of development, the banking industry is bound to face the bottlenecks such as redundant institutions, increasing operating costs, and declining operating income. In addition, users’ demand for online, personalized, and contactless services has been stronger amid the COVID-19 pandemic. Therefore, contactless financial services demonstrate the achievements of deepening digital transformation in the banking industry and an inevitable choice for banks to carry out the strategic transformation that can improve quality and efficiency. At present, contactless financial services are significant as they help the banking industry adapt to the impact of the pandemic in the short term and seek new drivers of business growth in the medium and long term. These services are now being integrated into enterprises’ production and people’s lives at an unprecedented speed, breadth, and depth, injecting new momentum into the real economy and people’s lives.

It can be seen from the development of contactless financial services in China’s banking industry in recent years that some pioneers in the field of digital transformation have used digital technology and financial means to innovate services before the pandemic and realized low-contact or even contactless financial services (Ba and Liu, 2020). After years of exploration and technological investment in the banking industry, contactless financial services have shown some problems while making a progress. For example, many banks simply regard contactless financial services as a supplement to offline services, and the small range of products and services fails to meet customers’ various needs. As a result, customer stickiness is not high. Meanwhile, the model of contactless financial services has weakened the environment to implement risk management policies, laws, and regulations based on traditional offline banking outlets, which threatens users’ privacy and property security, causes a trust crisis, and hinders the further development of contactless financial services.

Contactless financial services are an innovative exploration of the banking industry to integrate digital technology. Existing studies in this field are mainly focused on the content and patterns of the services, and the use behavior of contactless financial services has been rarely investigated. In response to this situation, this study focused on the use behavior of contactless financial services. It constructed a theoretical model based on the UTAUT model and trust theory and conducted an empirical study using data collected through questionnaires in order to provide relevant suggestions for the improvement of contactless financial services, promote use behavior, and boost the further development of contactless financial services.

## 2. Literature review and hypothesis development

### 2.1. Perceived risk and trust

The concept of perceived risk, initially deriving from psychology, was proposed by Bauer (1960). The theory refers to the extent to which the loss caused by the use of a certain new technology or product deemed by the users affects their acceptance of the technology or product, involving psychological, economic, and temporal aspects, among others. The perception of perceived risk is the core that influences users’ intention to pay.

Information systems are vulnerable and potential risks are inevitable, so security has been an ever-present issue since the creation of the Internet. When users decide whether to adopt an innovation, they evaluate it through their own or others’ experience and adopt risk-reducing strategies to avoid high-risk services (Cox and Rich, 1964). Research has shown that users’ perceptions of risks will influence their purchasing decisions (Park et al., 2005) and technology adoption (Lu et al., 2005; Martins et al., 2014). According to studies on e-commerce, perceived risks have a negative and significant effect on consumers’ willingness to purchase online (Wingate, 2019). Similarly, studies on e-payments have found that perceived risks have a negative effect on the use of online payments (Chang, 2018).

In this study, perceived risk refers to the user’s perception of adverse consequences due to uncertainty when using contactless financial services. Compared with traditional face-to-face purchases and payments, contactless financial services are mainly remote purchases of products or receipts of services, during which users may perceive the risks of exposing personal information, personal financial losses, and increasing unspecified costs. As a result, their intentions to use contactless financial services is reduced (Ba and Liu, 2020).

Trust can motivate human initiative and action consciousness. In the era of the Internet economy, since there is no direct communication and interaction between the buyer and the seller, and the consumer trust in products and services will increase the trend of purchase and use, trust has become the focus on academics (Larasetiati and Ali, 2019; Wu and Chen, 2020). Trust is the foundation of all commercial activities and behaviors and has become a focal point in the academic community in the era of a booming Internet economy as it can mobilize people’s agency and consciousness of action (Wu and Chen, 2020). In the theory of reasoned action (TRA) established by Fishbein and Ajzen (1975), trust, as a behavioral intention, is often used by consumers as a tool to reduce uncontrollability, uncertainty, and transaction costs in decision-making in risky environments. According to the basic attitude-intention-behavior framework, consumers establish trust beliefs to minimize perceived risk, which influences individual trust attitudes and ultimately affects the intention to use a service or product and behavioral outcomes.

The relationship between trust and purchase intention, Liu and Guo (2017) indicated that trust positively affects purchasing intentions. Di Zhao et al. (2019) also found customers have a sustainable purchase when they trust the seller and trust is an important factor in increasing interest in online shopping (Pappas, 2018). Studies have shown that trust is an important factor in user adoption of new technologies and products. In the study on the adoption of mobile banking, Zhou (2011) predicted that trust is the initial factor for users to adopt mobile banking and confirms that trust is the key factor for users to decide whether to use mobile banking. In addition, Hanafizadeh et al. (2014) revealed that trust and perceived trustworthiness are the key drivers for customers of Iranian banks to adopt mobile banking; Jiang et al. (2020) also pointed out that trust plays a positive role in users’ choice to use online consumer credit products.

In this study, trust refers to the extent to which users trust the information, products, and operational mechanisms provided by contactless financial services. With a growing trend of online transactions, many financial institutions (e.g., banks, securities, etc.) regard contactless platforms as important vehicles for publicity and marketing. In such a context, it is important for contactless financial services to provide reliable and objective information and products and operate a robust transaction mechanism to gain users’ trust. Trust affects users’ intention to use a product or service (Xu, 2021). Therefore, the following hypotheses were formulated.

*H1*: Perceived risk will have a negative effect on users’ trust.

*H2*: Perceived risk will have a negative effect on users’ behavioral intention.

*H3*: Trust will have a significant effect on users’ behavioral intention.

### 2.2. Unified theory of acceptance and use of technology

The Unified Theory of Acceptance and Use of Technology (UTAUT) was proposed by Venkatesh based on eight established information adoption models such as the Technology Acceptance Model (TAM) and the theory of planned behavior (TPB) (Venkatesh et al., 2003). In UTAUT, constructs of the eight models are integrated into four core constructs, performance expectancy, effort expectancy, social influence, and facilitating conditions, which explain users’ adoption behavior of information technology. Since its introduction, the UTAUT model has been used to analyze users’ intention to accept and use emerging information technologies, such as instant messaging software and mobile banking. Empirical tests have demonstrated that the UTAUT model has a 70% explanatory power for individual use behavior, better than the TAM model and the TRA model (Li and Yuan, 2020; Chen and Liang, 2021). The UTAUT model is the most used model with the best reliability in the academic community. Currently, it has been applied in several fields and has been proven to have great explanatory power. For example, Chao (2019) used the extended UTAUT model to study the behavioral intention of use mobile learning. Rashmini et al. (2020) used the UTAUT model to examine the adoption behavior of users in developing countries for mobile online banking. Alajmi and Alotaibi (2020) used the UTAUT model to study the intention to use mobile libraries. According to the analysis of studies from various countries, the UTAUT model greatly varies in terms of the significance of variables’ effects and applicability in different situations. To solve the problem, case-by-case analyses are needed to identify the key influencing factors in different application situations (Li and Yuan, 2020). Li and Min (2021) used the UTAUT model and CCC moderating effect to study the continued intention to use Moocs. Contactless financial services, as a new type of comprehensive financial services, change the traditional way of financial institutions to provide services with the help of physical outlets and a simple vehicle of voice, and they are different from the traditional mode in terms of the content and forms of services. Therefore, users’ intention and behavior to use the emerging contactless financial services may be explained by the UTAUT model to a certain extent, and the UTAUT model is used as the theoretical basis for this study.

According to the UTAUT model, performance expectancy, effort expectancy, and social influence affect behavioral intention and then the actual use behavior, while facilitating conditions directly impact use behavior. In the UTAUT model, performance expectancy is defined as users’ expectation that technologies and products can improve individual performance (Venkatesh et al., 2003). In this study, performance expectancy emphasizes the extent to which users recognize the improvements and enhancements brought by contactless financial services to their personal lives, work, etc. Effort expectancy refers to the extent to which users can easily accept new products and technologies, which in this study refers to the degree of difficulty perceived by users when using contactless financial services. Social influence denotes the degree of influence exerted by the surrounding groups perceived by users when using new technologies. Users’ intention to use contactless financial services might be influenced if their friends, relatives, and those who have a great influence on them are using these services.

Facilitating conditions refer to the extent to which users believe that the existing organization and technological structures can support the use of contactless financial services. However, with the popularity of intelligent communication devices, it has become much easier for users to obtain and use devices that support online consultation. In other words, as mobile intelligent devices have been widely used, the UTAUT is adjusted in this study according to the current situation, in which “facilitating conditions” is no longer used as a main factor influencing use behavior (Xu, 2021). Therefore, the following hypotheses were formulated.

*H4*: Performance expectancy will have a significant effect on users’ behavioral intention.

*H5*: Effort expectancy will have a significant effect on users’ behavioral intention.

*H6*: Social influence will have a significant effect on users’ behavioral intention.

*H7*: Users’ behavioral intention will have a significant effect on users’ use behavioral.

In the research system of trust in e-commerce, when consumers think that their needs match the expected performance of a product, their satisfaction will increase and the uncertainty in decision-making will decrease, which helps them to build trust in the product, having a positive impact on consumers’ behavioral intentions (Jiang et al., 2020). Martin et al. (2015) verified that users’ expectations of products and technologies in different decision-making situations have significant effects on the formation of individual trust. Meanwhile, in the research field of sociology, from the perspective of social relations and social culture, the building of trust in a particular object is influenced by cultural norms. In other words, social influences have effects on individual trust (Jiang et al., 2020). Currently, it has been proved that the opinions of people around an individual can affect individual trust (Yang et al., 2016). Therefore, the following hypotheses were formulated.

*H8*: Performance expectancy will have a significant effect on trust.

*H9*: Social influence will have a significant effect on trust.

### 2.3. Perceived advantage

Perceived advantage refers to the relative advantage of an information system in terms of information content, functional services, and system interface as perceived by users compared with other types of information systems (Wrycza et al., 2017). As Internet technologies continue to integrate with other industries, the comparison of advantages between channels has attracted academic attention. Researchers have studied the advantages of new channels in the context of the Internet perceived by users and proved that perceived advantages have a positive impact on user’s intention to use a product or service (Rosenzweig and Roth, 2007; Wu et al., 2019; Huang et al., 2020). In an earlier qualitative study, Lee et al. (2003) pointed out that the perceived advantage plays a significant role in improving users’ attitudes toward mobile banking. Similarly, Püschel et al. (2010) claimed that Brazilian customers’ attitudes toward mobile banking are significantly influenced by perceived advantage. And Lin (2011) also found that perceived advantage plays a key role in driving attitudes toward banking and ultimately leading users to adopt mobile banking.

In this study, contactless financial services are innovative channels for financial services. If users perceive that contactless financial services have advantages over traditional offline channels and the simple channels of customer service centers in terms of efficiency, products, and services, their intention to use contactless financial services may be boosted. Therefore, the following hypothesis was formulated.

*H10*: Perceived advantage will have a significant effect on users’ behavioral intention.

## 3. Research methodology

### 3.1. Research model

**Figure 1:**
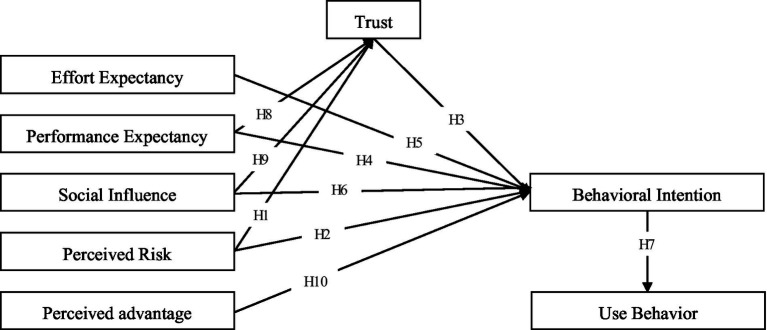
Theoretical model.

Based on the above analysis, this paper built a research model based on the UTAUT model. Eight constructs, namely performance expectancy, effort expectancy, social influence, trust, perceived risk, perceived advantage, behavioral intention and use behavior were selected based on the theories of perceived risk, trust, and perceived advantage. The model was used to explore the behavior of using contactless financial services (Figure 1).

### 3.2. Measure instrument

**Figure 2:**
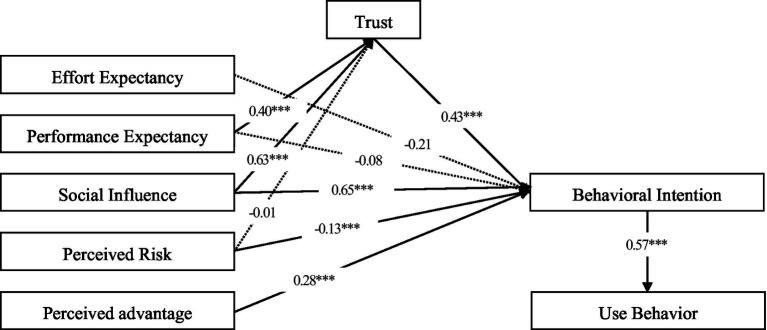
Path coefficients for the research model. ***p < 0.001.

Following the above analysis, eight constructs are included in the conceptual model. All of the constructs are measured by multi-item scales adapted from prior studies with minor change in wording in order to fit contactless financial services’ characteristics (as shown in the Appendix). This study drew on the studies by Venkatesh et al. (2003), Jiang et al. (2020), and Rashmini et al. (2020) to measure performance expectancy, effort expectancy, social influence, behavior intention, and use behavior, drew on the studies by Bauer (1960), Fishbein and Ajzen (1975), Wu and Chen (2020), and Xu (2021) to measure perceived risk and trust, drew on the studies by Wrycza et al. (2017), and Huang et al. (2020) to measure perceived advantage. A five-Likert scale, from strongly disagree to strongly agree, is adopted to measure the eight constructs. At last, in order to ensure the logical consistency and ease of understanding of the questionnaire, we sent 32 questionnaires to peer experts for pilot test to refine the questionnaire wording before formal data collection. On the whole, the questionnaire was unambiguous and easy to complete (Figure 2).

### 3.3. Questionnaire design and data collection method

The questionnaire method was used to collect users’ subjective data. The questionnaire consists of three parts: introduction, personal information collection, and the scale. The introduction part briefly illustrates the purpose of this study and matters such as the protection of users’ private information and explains the screenshots of the pages of contactless financial services provided by several representative banks (e.g., Industrial and Commercial Bank of China, China Minsheng Bank, Bank of Communications, etc.). The users filled in the questionnaire after reading the above content. The part of users’ demographic characteristics collected information about gender, age, education, etc. The scale part collected information about the eight constructs, namely performance expectancy (PE), effort expectancy (EE), social influence (SI), behavioral intention (BI), trust (TRU), use behavior (UB), perceived risk (PR), and perceived advantage (PA).

To ensure that the respondents cover a comprehensive range of people and are representative, the population aged between 18 and 65 was selected as the main respondents, taking into account the actual situation of the use of contactless financial services and mobile communication devices. Electronic questionnaires were distributed *via* a questionnaire website, and 411 valid questionnaires were collected from 7 September to 30 September 2022.

### 3.4. Demographic

The 2021 White Paper on Digital Banking in China> shows that younger users have weaker preferences for offline banking outlets and that more than half of digital banking users have a bachelor’s degree or higher (iResearch, 2021). According to the statistics on the characteristics of the sample, males and females accounted for 53.5 and 46.5%, respectively, of the respondents, almost evenly distributed. The age groups of 18–30, 31–50, and 51–65 made up 58.7, 36.2, and 5.1%, respectively. In terms of education, 72.8% had a junior college or bachelor’s degree, and 14.8% had a master’s degree or above. In terms of occupation, the proportion of government officials and employees of enterprises and public institutions was 62.3%. In terms of the frequency of use, 29.9% of the respondents use these services 1–3 times a year, 58.9% use them 3 to 5 times a year, and 11.2% use them more than 5 times a year. In terms of the preference for financial service channels, 75.7% of the respondents preferred contactless channels, and 24.3% preferred offline channels. In addition, 81.5% of the respondents said they knew contactless financial services. The sample data were consistent with the actual situation, with representativeness and diversity, meeting the requirements of the study for sample characteristics.

## 4. Results of statistical analysis

The structural equation modelling (SEM) approach was used to validate the research model. We analyzed the generated hypotheses by using AMOS version 23.0. Based on the two-step approach recommended by Anderson and Gerbing (1988), this study first analyzed the measurement model to test the reliability and validity of the instrument, and then analyzed the structural model to test our research hypotheses (Bentler, 1989).

### 4.1. Measurement model analysis

To determine the reliability of each item, this study examined the significance and magnitude of its loadings. As shown in Table 1，all items loaded significantly on their respective latent factors, and they all achieved standardized loadings of at least 0.60. Two measures were used to assess the internal consistency of the constructs: composite reliability (CR) and average variance extracted (AVE). The CRs were in the range between 0.77 and 0.91, and the AVEs were in the scope from 0.56 to 0.67; therefore, all were above the recommended cut-off levels of 0.70 and 0.50, respectively, and revealed good internal consistency. In the validity analysis, the convergent validity was also examined through composite reliability.

**Table 1:** Loadings and composite reliability.

Variables	Items	Loadings	Cronbach’s *α*	AVE	CR
Performance expectancy	PE1	0.79	0.89	0.57	0.80
	PE3	0.68			
	PE5	0.79			
Effort expectancy	EE1	0.80	0.83	0.62	0.77
	EE3	0.78			
Social influence	SI1	0.77	0.85	0.57	0.84
	SI2	0.73			
	SI3	0.73			
	SI4	0.79			
Perceived risk	PR1	0.83	0.91	0.67	0.91
	PR2	0.86			
	PR3	0.82			
	PR4	0.77			
	PR5	0.82			
Perceived advantage	PA1	0.78	0.87	0.56	0.79
	PA2	0.70			
	PA3	0.75			
Trust	PB1	0.79	0.92	0.63	0.89
	PB2	0.83			
	PB3	0.79			
	PB4	0.81			
	PB5	0.75			
Behavioral intention	BI2	0.90	0.84	0.67	0.80
	BI4	0.73			
Use behavior	UB1	0.71	0.83	0.66	0.79
	UB3	0.90			

The discriminant validity was measured by examining the average variance extracted (AVE). According to Fornell and Larcker (1981), the discriminant validity is demonstrated because the correlations between constructs are all below the square root of the AVE per construct. As shown in Table 2, no correlation coefficients between constructs exceed the square root of the AVE, which supports a satisfactory discriminant validity.

**Table 2:** AVEs and correlation coefficients of constructs.

	1	2	3	4	5	6	7	8
1.Performance expectations	0.76							
2. Effort expectancy	0.65	0.79						
3. Social influence	0.68	0.73	0.76					
4. Perceived risk	0.39	0.45	0.53	0.82				
5. Perceived advantage	0.69	0.64	0.72	−0.42	0.75			
6. Trust	0.64	0.57	0.67	−0.49	0.57	0.79		
7. Behavioral intention	0.71	0.68	0.69	−0.39	0.72	0.58	0.82	
8. Use behavior	0.55	0.69	0.68	−0.42	0.71	0.61	0.79	0.81

Diagonal figures are square root of AVE. *p, 0.05; **p, 0.01; ***p, 0.001.

### 4.2. Structural model analysis

As the measurement model was acceptable, hypothesized relationships were tested by estimating the statistical significance of path coefficients for the structural model. From Table 3, it is found that *χ*2/df is 2.56, TLI value of 0.94，GFI value of 0.95, RFI value of 0.91, NFI value of 0.93, IFI value of 0.95, and RMSEA value of 0.06. Therefore, the model has a good fit.

**Table 3:** Validity factor analysis model fit.

Index name	χ^2^/df	TLI	CFI	RFI	NFI	IFI	RMSEA
Index criteria	<3	>0.9	>0.9	>0.9	>0.9	>0.9	<0.08
Actual value	2.56	0.94	0.95	0.91	0.93	0.95	0.06

Table 4 shows the results in the model testing is to examine the path significance of each hypothesis. The results showed that hypotheses H2, H3, H6, H7, H8, H9, and H10 had absolute CR values greater than 1.96 and *p*-values less than 0.05, indicating that they passed the significance test. Therefore, these seven hypotheses were supported. H1, H4, and H5 did not pass the significance level test and were thus not supported (Figure 2).

**Table 4:** Hypotheses testing results.

Path	Standardized path coefficient	S.E.	C.R.	*p*
H1: TRU ← PR	−0.01	0.03	−0.36	0.72
H2: BI ← PR	−0.13	0.04	−2.97	***
H3: BI ← TRU	0.43	0.10	4.25	***
H4: BI ← PE	−0.08	0.12	−0.62	0.53
H5: BI ← EE	−0.21	0.11	1.84	0.07
H6: BI ← SI	0.65	0.11	5.89	***
H7: UB ← BI	0.57	0.10	5.49	***
H8: TRU ← PE	0.40	0.06	6.49	***
H9: TRU ← SI	0.63	0.07	8.90	***
H10: BI ← PA	0.28	0.07	3.95	***

*p ≤ 0.05; **p ≤ 0.01; ***p ≤ 0.001.

## 5. Discussion

This study further modified the UTAUT model based on the theories of trust, perceived risk, and perceived advantage and constructed a conceptual model to examine the influencing factors of the behavior of using contactless financial services. The model was validated using the data collected through questionnaires. The following conclusions were drawn.

The standardized path coefficient between perceived risk and behavioral intention was −0.13, and *p* < 0.005, indicating that perceived risk negatively influences the behavioral intention. Therefore, H2 was supported. The standardized path coefficient between trust and behavioral intention was 0.43, and *p* < 0.001, indicating that trust positively influences the behavioral intention. Therefore, H3 was supported. The findings are consistent with previous studies (Liu et al., 2020). When users have greater concerns about adverse consequences due to uncertainty when using contactless services, the use of this type of service is somewhat hindered. Contactless financial services in China have witnessed a rapid development since the pandemic. In order to block the transmission of the pandemic and protect citizens, the government has implemented several preventive and control measures. For example, public places were forced to close down. Therefore, business that needed to be handled on offline scenarios was switched to contact-free channels. In this context, many financial institutions, led by the banking industry, have made digital transformation and have completed channel conversion in a short time. However, at that time, risk management measures, laws and regulations related to contactless financial services were not perfect enough, which brought many potential risks. Lawbreakers also took advantage of the chaotic period of transformation to commit frauds, resulting in an increase in financial crime rate. Now that users needed to face increased risk of uncertainty when using contactless financial services, users were less willing to use the services in order to avoid relevant risks. Trust has a significant positive impact on the behavioral intention. Users show a greater intention to use contactless financial services when they have higher recognition of the validity of products and service information, user privacy protection, transaction environment, and security building provided by this type of service. This result is consistent with the conclusion about the relationship between trust and the behavioral intention drawn in the study on the impact of trust on users’ online purchasing behavior by Wu et al. (2020), indicating that trust is also one of the important factors influencing use.

Both social influences and perceived advantages positively influence the behavioral intention, with standardized path coefficients of 0.65 and 0.28, respectively, and *p* < 0.001. Therefore, H6 and H10 were supported. It can be seen that users’ intention to use something is greatly influenced by people around them and the social environment, which is consistent with the conclusion about social influence and the behavioral intention drawn in the study on users’ acceptance of new technologies by Kumar et al. (2016). Users consider contactless financial services advantageous as more businesses can be dealt with online, and traditional offline channels have some problems such as a long waiting time and low efficiency. As a result, the overall satisfaction with contactless channels is significantly higher than that of traditional outlets. At present, as the pandemic still lingers, the use of contactless services not only meets users’ demand for less contact but also conforms to the trend of social development. These services can improve user stickiness and have great potential for future development.

The standardized path coefficient between the behavioral intention and the use behavior was 0.57, and *p* < 0.001, indicating that the intention positively influences use behavior. Therefore, H7 was supported. This conclusion is consistent with that drawn by Venkatesh et al. When users show a great intention to use contactless financial services, they are more motivated to translate ideas and plans into action. Meanwhile, <the 2021 White Paper on Digital Banking in China>shows that most users have expectations for contactless financial services, and they are willing to use and recommend this type of service.

The standardized path coefficient between performance expectancy and trust and that between social influence and trust were 0.40 and 0.63, respectively, and *p* < 0.001. Therefore, H8 and H9 were supported. It indicates that during the process of building trust in contactless financial services, users are greatly affected by their expectations for related products and services, as well as by people around them and sociocultural norms. The advancement of Internet technology has promoted the development of social media and virtual communities online. As users’ feelings about contactless financial services are spread through interpersonal relationships and social networks, positive comments on this type of service can strengthen users’ trust.

The standardized path coefficient between perceived risk and trust was −0.01, and *p* = 0.72. Therefore, H1 was not supported, which is not consistent with previous studies. This is closely related to the particular context of the pandemic. The public spaces were closed under the pressure of the pandemic. The Chinese government launched policies to guide the banking sector to actively develop the contact-free finance. And educate the public on cyber security *via* official media accounts. According to the statistics of the China Banking Association, the market share of non-contact services of banking institutions reached 96% on average during the pandemic. It can be seen that under the sociocultural environment where the government and banks actively conduct public education and guidance, the public trust the government and banks at a high level. And are willing to believe that the products and services provided by the non-contact channel are safe, reliable and can be used for daily business needs. As digital technology develops, new types of network fraud frequently occur. To solve the problem, various departments actively spread relevant knowledge of network security and strengthen the protection of users’ personal information and property security. In addition, users’ network security awareness has been greatly enhanced as they have more experience in online behavior and contactless services. In such a context, they believe that their rights and interests are protected when using contactless financial services.

The standardized path coefficient between performance expectancy and the behavioral intention and that between effort expectancy and the behavioral intention were −0.078 and −0.21, respectively, and the *p*-values were 0.53 and 0.07, respectively. Therefore, H4 and H5 were not supported. During this special period of the pandemic, many offline venues shut down. The purpose of users is not to enhance their personal technology or performance through the use of contactless financial services but to handle necessary business in order to maintain the normal process of their work and life. Therefore, the influence of performance expectation on the willingness to use is not significant. In recent years, digital banking has been upgraded in three main aspects: system support, data management and user perception. Due to the pandemic, the banking industry attaches more importance to the construction of contactless financial business, allowing users to enjoy faster, simpler and more comprehensive contactless financial services, thus greatly improving the stability and usability of the contactless financial services system. According to the Statistic Report of on China’s Internet Development, as of June 2022, the Internet penetration rate in China reaches 74.4%, with 99.6% of netizens using mobile devices. Through the analysis of the characteristics of the samples in this study, the preference users of contactless financial services are younger, and have higher education and better digital literacy, which greatly reduces their perceived technical difficulties when using contactless financial services. In recent years, digital banking has been upgraded mainly in three dimensions, namely system support, data management, and user perception. As users who prefer contactless channels are relatively young, it is not difficult for them to use digital technologies and communication devices. At the same time, as they are used to solving problems through online channels, the perceived usefulness and efficiency are greatly reduced.

## 6. Conclusion

This study analyzed the influence mechanism of the behavior of using contactless financial services and made the following suggestions on how to develop contactless financial services and increase users’ intention to use these services, in a bid to accelerate the rollout of contactless financial services and enhance their influence.

Further improving the ability to collect and integrate information and providing personalized services. Platforms offering contactless financial services should follow the development of digital technology, make full use of 5G, big data, cloud computing, and other technologies to further improve the ability to collect mass data and conduct data mining, and establish user profiles. Based on the analysis of user profiles, personalized content recommendations are integrated to meet user needs and improve the efficiency of users’ acquisition of information.

Laws and regulations in contact-free scenarios should be improved to create a safe transaction environment. On the one hand, relevant government departments need to improve the relevant laws and regulations governing transactions and services conducted on the basis of contact-free scenarios as soon as possible, in order to clarify the rights and obligations of both parties to the transactions. On the other hand, through the introduction of block chains and other technologies, the risk prevention and control capability of non-contacted scenarios shall be enhanced, to reduce system risks and strengthen the stability of system operation.

It is necessary to establish a comprehensive mechanism to protect the user privacy and increase user willingness to use contactless financial services. In the process of users involving in contactless financial services, the collection and use of users’ information by service providers should be severely regulated. By ensuring the openness and transparency of information collection and use, and effectively protecting user information and property security, service providers can reduce cyber risks and increase users’ confidence in using the service.

Refining the policies and regulations in the digital environment and creating a safe transaction environment. On the one hand, relevant government departments should accelerate the formulation of laws and regulations related to financial products and services on the Internet to protect the legitimate rights and interests of users while spreading knowledge about network fraud and stepping up users’ precautions. On the other hand, platforms offering contactless financial services can strengthen the protection of users’ information by introducing technologies such as blockchain to reduce users’ risks while ensuring strict compliance with transaction service commitments during operations.

Relevant government departments and financial institutions should promote and guide the rollout of contactless financial services in a planned and organized manner. Nowadays, China is vigorously promoting digitization, and the development of contactless financial services is in line with the national demand. Against such a backdrop, it is advisable to make the most of the favorable policy environment, strengthen the promotion and publicity of enterprises, focus on brand building, and optimize the effects to continuously improve users’ trust in contactless financial services and their intention to use these services. Meanwhile, as large numbers of young are using contactless financial platforms, the platforms should further simplify the interface settings and operation processes to meet the needs of different people and enhance the influence of their services.

This study has some shortcomings. First, in terms of the measurement of the behavioral intention and use behavior, four items were initially designed to measure the two constructs, but only two were retained given factors such as the factor analysis and Cronbach’s alpha. With the advancement of digital technologies, the increasing popularity of mobile devices, and the improved digital literacy of users, it has become much easier to download and use contactless services. All these factors have resulted in an unclear understanding of the differences between the two constructs. Future research should explore the measurement scales for the two constructs in the digital environment. Second, in the age distribution of the samples, young people aged 18–30 account for nearly 60%, and they are the main users of non-contact services. At present, with the strategic goal of building digital China strategy and national cyber development strategy, diverse industries are speeding up the pace of digital transformation. Digital technologies penetrate every aspect of people’s life. However, there is a huge gap in the digital literacy and technological level of different regions and groups in China. In order to further expand the audience and impact of contact-free services, differentiated and digitally inclusive services should be provided according to the specific needs of different groups. Finally, as the pandemic situation eases, life and work order is gradually restored, but in the process of fighting against COVID-19, the way of human life and work has been already changed, and the non-contact economy has emerged as a dark horse. In the post-pandemic era, the contact-free economy will continue to exist as an economic innovation. It will also play an important role in the transformation of industrial and economic structures across the globe. Therefore, we should continue to pay attention to the development of new technologies and users’ technology adoption behavior in the contactless economy in the future.

## Data availability statement

The original contributions presented in the study are included in the article/supplementary material, further inquiries can be directed to the corresponding author.

## Author contributions

LC constructed the theoretical framework and research model for this study. JJ designed the questionnaire and completed the data analysis. CW summarized the references and made recommendations. All authors approved the final version of the manuscript for submission.

## Conflict of interest

The authors declare that the research was conducted in the absence of any commercial or financial relationships that could be construed as a potential conflict of interest.

## Publisher’s note

All claims expressed in this article are solely those of the authors and do not necessarily represent those of their affiliated organizations, or those of the publisher, the editors and the reviewers. Any product that may be evaluated in this article, or claim that may be made by its manufacturer, is not guaranteed or endorsed by the publisher.
